# Cytokine hemoadsorption with CytoSorb® in patients with sepsis: a systematic review and meta-analysis

**DOI:** 10.5935/2965-2774.20230289-en

**Published:** 2023

**Authors:** Jiovany Jhan Carlos Saldaña-Gastulo, María del Rosario Llamas-Barbarán, Lelis G. Coronel-Chucos, Yamilée Hurtado-Roca

**Affiliations:** 1 Universidad Científica del Sur - Lima, Peru; 2 Hospital Nacional Dos de Mayo - Lima, Peru

**Keywords:** Cytokine, Hemoperfusion, Mortality, Sepsis, Septic shock

## Abstract

**Objective:**

To analyze the effect of CytoSorb® on mortality, interleukin levels, vasopressor use and adverse events in patients with sepsis.

**Methods:**

We searched MEDLINE®, Embase and the Cochrane Library for randomized controlled trials and cohort studies that reported the use of CytoSorb® among septic patients. The primary outcome was mortality, and secondary outcomes included the use of vasopressors, levels of inflammatory markers, predicted *versus* observed mortality, length of stay in the intensive care unit, and adverse events.

**Results:**

We included 6 studies enrolling 413 patients, and assessment for risk of bias indicated variations in study quality from high to moderate. The overall mortality rate was 45%, and no significant effect on mortality was found at 28 - 30 days (RR 0.98 [0.12 - 8.25] for the randomized clinical trial and RR 0.74 [0.49 - 1.13] for cohort studies). We did not perform a metanalysis for other outcomes due to the small number of studies found or the lack of data.

**Conclusion:**

Our study found very low certainty evidence, due to imprecision, risk of bias, and heterogeneity, thereby showing no benefit of CytoSorb® use in terms of mortality at 28 - 30 days. We cannot recommend the use of CytoSorb® in septic or septic shock patients outside clinical trials. Further high-quality randomized trials with a common intervention arm are needed to evaluate the influence of CytoSorb® in this population.

**PROSPERO register:**

CRD42021262219

## INTRODUCTION

Sepsis is defined as life-threatening organ dysfunction caused by a dysregulated host response to infection,^(1)^ and its most severe state, septic shock, represents a highly lethal condition that causes substantial morbidity and mortality among critically ill patients.^(2)^ The pathophysiology of sepsis is very complex, involving many factors, such as proinflammatory and anti-inflammatory cytokines, pathogen-associated molecular patterns (PAMPs), bacterial exotoxins and endotoxins, mycotoxins, damage-associated molecular patterns (DAMPs) released by injured cells and host-specific factors such as activated complement and procalcitonin.^(3)^ Inflammation can lead to severe immune system dysfunction ranging from destructive maladaptive systemic inflammatory response syndrome (SIRS) to advanced immunosuppression, which could lead to multisystem organ dysfunction and death.^(3,4)^

Despite early treatment and multiple efforts to reduce mortality in sepsis and septic shock, such as the surviving sepsis campaign, which provides treatment guidelines,^(5)^ mortality is still high, approximately 20 - 40% for severe sepsis and 40 - 60% in septic shock,^(1,6,7)^ without significant variations in this figure in recent years. This is why adjuvant therapies, such as blood purification techniques including extracorporeal removal of cytokines by hemoadsorption, have been described.^(2,8-10)^

There are currently multiple blood purification techniques, with different results, such as cytokine removal, decrease in vasopressors and even decrease in mortality; such techniques include high-volume dialysis, high-cut membranes, adsorption by filtration coupled plasma and special adsorption filters (such as Oxiris, CytoSorb®, HA 330 and Polymyxin B filters).^(8,11)^ Blood purification therapies have been used in different acute inflammatory scenarios, such as sepsis, cardiac surgery, and autoimmune diseases; however, their use is controversial, and despite a theoretical justification, the use of blood purification methods cannot yet be recommended for patients with sepsis due to a lack of evidence.^(12)^

CytoSorb® is a cartridge composed of polystyrene-divinyl-benzene polymer beads with a highly porous and biocompatible polyvinylpyrrolidone cover. Its estimated size is 300 to 800µm with a total surface area of more than 40,000m^2^. The elimination of substances from the blood is based on the capture of substances in the pores and surface adsorption. The typical duration of therapy is up to 24 hours per session, daily for 2 to 7 consecutive days.^(8,13)^ The physiological reason for using CytoSorb® in the setting of sepsis is to restore a balanced response of pro-inflammatory and anti-inflammatory mediators. Elevated circulating concentrations of several cytokines, including TNF-α, IL-1β, IL-6, IL-8, and IL-10, have been reported to be associated with morbidity and mortality in patients with sepsis, so their removal would be useful for treatment.^(3,11)^

We performed a systematic review and meta-analysis with the aim of analyzing the use of CytoSorb® in terms of mortality, interleukin levels, the use of vasopressors and adverse events in patients with sepsis and septic shock since the available evidence is still controversial.

## METHODS

A systematic review was conducted in accordance with the Preferred Reporting Items for Systematic Reviews and Meta-Analyses (PRISMA) statement,^(14)^ and a research protocol was developed and registered in the International Prospective Register of Systematic Reviews (PROSPERO) database (CRD42021262219).^(15)^

### Information sources and search strategy

We conducted a systematic search in the PubMed, Embase, and Cochrane Library databases. Medical Subject Headings (MeSH) or equivalent terms were used. Articles in English and Spanish were included. A preliminary search strategy was created for MEDLINE®/PubMed®, the other searches were tailored to individual databases (Table 1S - Supplementary Material), and the overall search was performed from inception to November 2022. Additionally, the bibliographic references of the selected articles were reviewed to identify other references relevant to the topic. The MEDLINE search strategy was developed as follows: (“CytoSorb”[tiab] OR “Cytokine adsor*”[tiab]) AND (Sepsis [Mesh] OR Sepsis [tiab] OR sept* [tiab]).

For the Embase and Cochrane Library databases, the search strategy was developed with the terms “CytoSorb”, “Cytokine adsorption”, “hemadsorption”, “Septic shock” and “Sepsis”.

### Study selection

Relevant studies were identified by 2 reviewers, who independently assessed them using the research objectives and question (PICO). When an agreement was not reached, a third reviewer member of the investigator group was included and resolved any discrepancies. The articles selected in each database were exported to Zotero software, where the elimination of duplicates was carried out.

We included studies that met the following criteria: adult patients with sepsis or septic shock; randomized clinical trial (RCT) studies, propensity score-matched cohort studies (prospective or retrospective), or studies with historical control; patients who received at least one hemoadsorption therapy with CytoSorb®; and studies that reported on mortality at 28-30 days, requirement for the use of vasopressors, inflammatory marker levels and adverse effects of CytoSorb® treatment. The exclusion criteria were as follows: use of CytoSorb® in contexts other than sepsis and septic shock (such as pancreatitis, cardiac surgery, endocarditis, transplant, trauma or coronavirus disease 2019 - COVID-19); type of study or publication of type reports of cases or letters to the editor. We also did not include abstracts from conferences or before and after studies without a comparator group; studies in neonates or pediatric patients; and studies that did not report mortality data. It was deemed appropriate to include nonrandomized studies of interventions (NRSI) due to the low number of clinical trials found according to the research question.

### Data extraction and risk of bias assessment

A standardized data extraction sheet was used. Two independent reviewers extracted the data, and disagreements were resolved by discussion and consensus in case no agreement was reached. A third reviewer was included to resolve discrepancies.

The following information was extracted: name of the main author, year of publication, journal of publication, place of study, inclusion and exclusion criteria, patient population, time of initiation of intervention use, CytoSorb® dose used, mean age, number of patients, general mortality rate and predicted mortality for the groups. Additionally, data on preand posttreatment changes in inflammatory markers and vasopressor levels were collected, if available.

Two authors performed the risk of bias assessment. We used the risk of bias tool (ROB) for the RCTs^(16)^ and the Review Manager 5.4 program (Review Manager; The Nordic Cochrane Centre, Copenhagen, Denmark). The risk of bias assessment tool for nonrandomized interventions (ROBINS I)^(17)^ was used for the cohort studies, as recommended by the Cochrane collaboration. Importantly, ROBINS-I bias assessments were made based on the comparison between a given study and a theoretical randomized controlled trial with an ideal design for the study question, which represented the standard for a “low risk study” (Tables 2 and 3 - Supplementary Material)

### Data synthesis and analysis

The outcomes were analyzed using the Mantel-Hansel statistical method and the Der Simonian-Laird random effects models, in relation to the high heterogeneity between the studies. The studies were not equivalent, they differed in the starting time, the duration of therapy, type of administration, and the source of sepsis, among other characteristics, which could have affected the results; therefore, a common effect size could not be assumed. Relative risks (RRs) for overall mortality, with 95% confidence intervals (95%CIs), were calculated for the conventional treatment and CytoSorb® treatment groups. Quantitative synthesis was not performed when only one study per outcome was identified or the studies were of a different type of design, or when the studies did not report the necessary statistics, which in that scenario were limited to a qualitative description.

The Review Manager 5.4 program was used for the analysis, and a p value < 0.05 was considered to indicate statistical significance.

Publication bias was not assessed due to the number of included studies.

## RESULTS

### Search results and study characteristics

**Figure 1:**
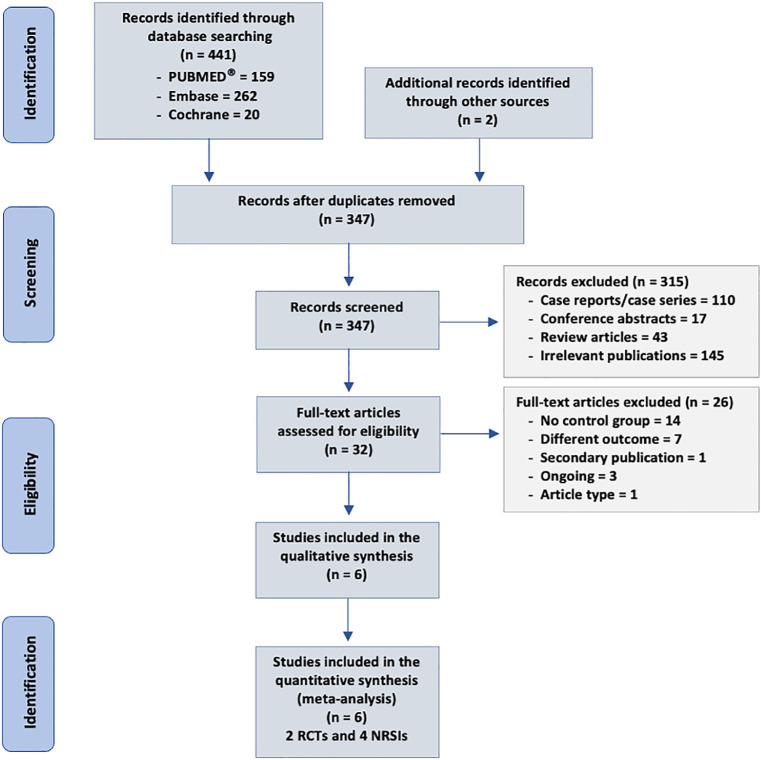
Flowchart of study selection.

Our search strategy identified 443 citations, of which 32 were judged to be potentially eligible based on titles or abstracts, or both, and the full texts were obtained. We excluded 26 articles after reviewing the full text: 14 for not having a comparator group, 7 for having a different outcome or mixed population, 3 for reporting studies in progress, 1 for being a different type of article, and 1 for being a secondary publication. Finally, 6 studies were included (2 RCTs and 4 cohort studies) including 413 patients.^(9-11,18-20)^Figure 1 shows our flow chart of study selection.

RCT - randomized controlled trial; NRSI - nonrandomized studies of interventions.

Patients in the included studies had different causes of sepsis and septic shock. In addition, they differed in the mode, starting time and number of CytoSorb® treatment sessions. Table 1 summarizes the characteristics of the included studies.

**Table 1:** Characteristics of the included studies

Study/country	Study design	Specific population	Control group	Time of first CytoSorb® initiation	Duration of CytoSorb® therapy	Mortality in CytoSorb® group (%)
Schittek et al.,^(9)^ Germany	Retrospective control group and prospective intervention group	Patients in severe septic shock with sepsis-associated acute kidney injury	Retrospective controls with septic shock (rising noradrenaline dose above 20µg/minute) with sepsis associated acute kidney injury in CVVHDF	No information	No information overall. Survivors, approximately one cartridge per patient was utilized as the median (IQR 1 - 2) for 35.5 hours (17 - 47)	76.70
Hawchar et al.,^(10)^, Hungary	RCT	Septic shock	Patients with septic shock of medical origin, on mechanical ventilation, norepinephrine > 10µg/minute, procalcitonin > 3ng/mL without the need for renal replacement therapy	Started within 24 hours after ICU admission	24 hours	0
Rugg et al.,^(11)^ Austria	Propensity-score-weighted retrospective study	Primary or secondary sepsis	Matched controls were treated for septic shock and required RRT but did not receive CytoSorb® therapy. A generalized propensity score and Mahalanobis distance matching method (‘genetic’ matching) was applied	Initiation of CytoSorb therapy varied from 0.5 to 719 hours after ICU admission, but most patients received treatment within the first days	1 - 6 x 24 hours without interruption	21.40
Akil et al.,^(18)^ Germany	Cohort historic control	Pneumogenic sepsis and ECMO	Historical cohort. Patients with pneumogenic septic shock accompanying acute respiratory failure, invasive hemodynamic monitoring, and demand for norepinephrine 0.3µg/minute; elevated lactate concentrations 2.0mmol/L; and procalcitonin serum level 1ng/mL were eligible	Within 6 hours after admission to our ICU	Minimum 2 x 24 hours without interruption	0
Brouwer et al.,^(19)^ The Netherlands	Propensity-score weighted retrospective study	Septic shock	Patients with septic shock treated with CRRT without CytoSorb®. Stabilized inverse probability treatment weight was applied	CytoSorb® was initiated at the discretion of the treating intensive care physician	24 hours, mean duration of 2.34 ± 0.16 days	52.20
Schädler et al.,^(20)^ Germany	RCT	Severe sepsis or septic shock within 72 hours of ARDS or acute lung injury	Mechanically ventilated patients with severe sepsis or septic shock in the setting of acute lung injury or acute respiratory distress syndrome established within the last 72 hours	Enrollment within 72 hours of diagnosis of sepsis with ARDS/ALI	Maximum 7 x 6 hours 24 hours apart	36

CVVHDF - continuous venovenous hemodiafiltration; IQR - interquartile range; RCT - randomized controlled trial; ICU - intensive care unit; RRT - renal replacement therapy; ECMO - extracorporeal membrane oxygenation; CRRT - continuous renal replacement therapy; ARDS - acute respiratory distress syndrome; ALI - acute lung injury.

### Risk of bias in the included studies

**Figure 2:**
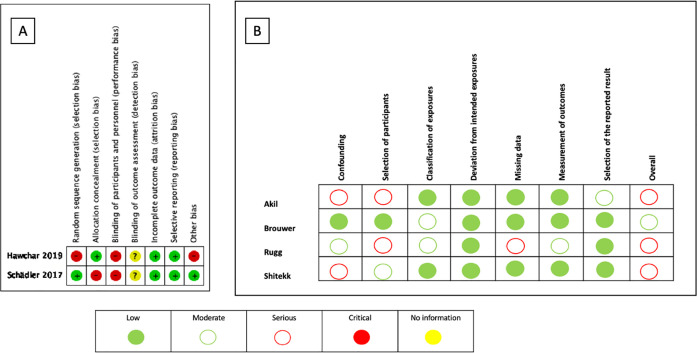
Risk of bias assessment of eligible studies. (A) Randomized controlled trial; (B) Nonrandomized studies of interventions.

Randomized clinical trials presented a high risk of bias; in none of them was it possible to blind the intervention for the outcome assessors. Cohort studies presented a moderate to severe risk. The risk of bias assessment is shown in figure 2.

### Effect on mortality at 28 - 30 days

**Figure 3:**
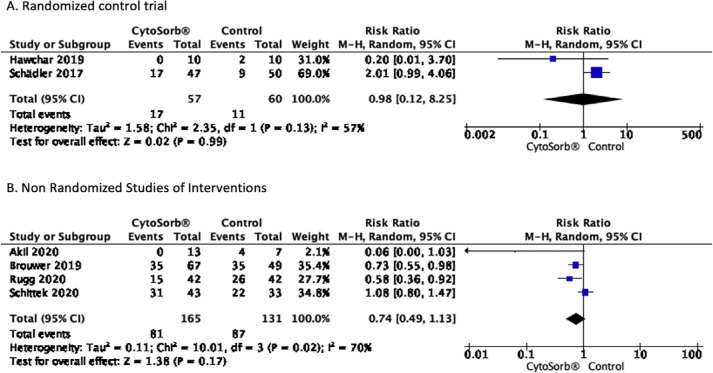
Forest plot effect of CytoSorb® on mortality at 28 - 30 days. (A) Randomized controlled trial; (B) Nonrandomized studies of interventions.

**Figure 4:**
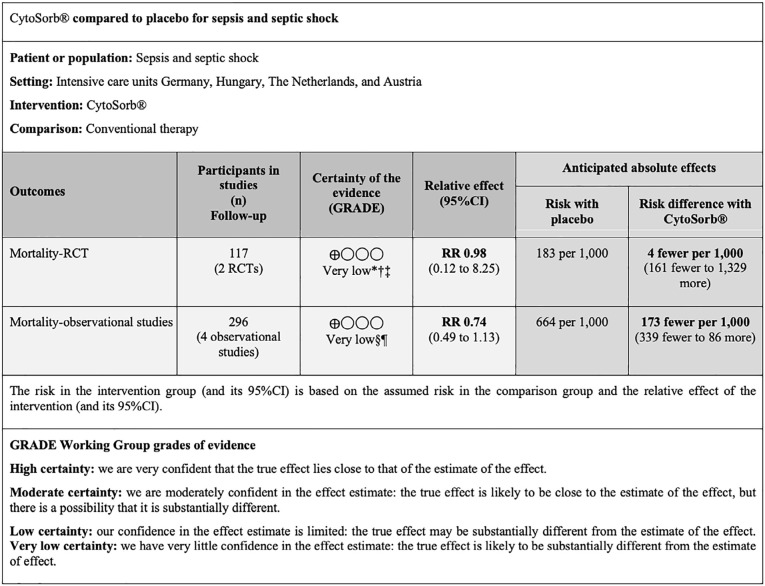
Summary of findings for the main comparison.

Overall mortality was 45% (42% intervention group and 48% control group),^(9-11,18-20)^ and only one study showed mortality greater than 70%.^(9)^ A quantitative review was carried out, finding no significant effect on mortality at 28 - 30 days RR 0.98 [0.12 - 8.25] for the RCT and RR 0.74 [0.49 - 1.13] for NRSI. The results are shown in figure 3, and a summary of the findings is shown in figure 4.

* It was decided to decrease two levels due to the high risk of bias; † it was decided to decrease one level due to the different results; ‡ it was decided to decrease two levels due to the wide confidence interval; § it was decided to decrease two levels due to the different results; ¶ it was decided to decrease two levels due to the wide confidence interval. GRADE - Grading of Recommendations Assessment, Development and Evaluation; 95%CI - 95% confidence interval; RCT - randomized controlled trial; RR - risk ratio.

### Effect on the use of vasopressors

Two studies, one RCT^(10)^ and one cohort study,^(18)^ reported the use of vasopressors as an outcome; both reported a significant decrease in vasopressor levels in the intervention group; however, this reduction was also shown in the control group (Table 2).

**Table 2:** Effect of CytoSorb® on secondary outcomes

	Control group			CytoSorb® group		
**Norepinephrine levels (ug/kg/min)**						
Study	NE T0	NE 48 hours	p value	NE T0	NE 48 hours	p value
Hawchar et al.^(10)^	0.43 [0.19 - 0.64]	0.25 [0.08 - 0.65]	NR	0.54 [0.20 - 1.22]	0.16 [0.07 - 0.48]	0.016
Akil et al.^(18)^	0.83 ± 0.16	0.38 ± 0.11	0.05	0.603 ± 0.08	0.009 ± 0.005	0.0001
**C-reactive protein levels (mg/L)**						
Study	CRP T0	CRP 48 hours	p value	PCR T0	PCR 48 hours	p value
Hawchar et al.^(10)^	307.4 ± 116.7	189.9 ± 48.5	NS	238.1 ± 95.5	169.54 ± 86.4	NS
Akil et al.^(18)^	27.2 ± 2.9	22.6 ± 3.1	0.31	35 ± 5	12 ± 3	0.002
**Procalcitonin levels (ng/mL)**						
Study	PCT T0	PCT 48 hours	p value	PCT T0	PCT 48 hours	p value
Hawchar et al.^(10)^	13.2 [7.6 - 47.8]	9.2 [3.8 - 44.2]	NR	20.6 [6.5 - 144.5]	5.6 [1.9 - 54.4]	0.004
Akil et al.^(18)^	13.14 ± 9.7	8.14 ± 5.9	0.68	15.6 ± 5.4	2.71 ± 1.5	0.03
**Predicted *versus* observed mortality**						
Study	Observed mortality	Predicted mortality	p value	Observed mortality	Predicted mortality	p value
Brouwer et al.^(19)^	51%	67,9%	0,035	47,8%	74,5%	< 0,001
Rugg et al.^(11)^	47,6%	65,7%	NR	21,4%	85,7%	NR

NE - norepinephrine; CRP - C-reactive protein; PCT - procalcitonin.

### Effect on levels of inflammatory markers

Only one study reported a 5 - 18% decrease in interleukin-6 (IL-6) levels;^(20)^ however, no statistical significance was found.

Two studies, one RCT^(10)^ and one cohort study,^(18)^ reported results for C-reactive protein (CRP). In the RCT, CRP levels did not show a significant difference; however, in the other study, a significant difference was found in the CytoSorb® group. These results are shown in table 2.

Regarding the PCT (procalcitonin) levels, two studies, one RCT^(10)^ and one cohort study,^(18)^ reported a significant decrease in PCT levels compared to the baseline level; nevertheless, this significant reduction was not found in the control group. These results are shown in table 2.

### Effect on predicted *versus* observed mortality

Two NRSI-type^(11,19)^ studies reported a decrease in observed mortality overpredicted mortality. Brouwer et al.^(19)^ 75% *versus* 52.2%, and Rugg et al.^(11)^ 85.1% versus 21.4%. In both studies, predicted mortality was calculated by the Sequential Sepsis-related Organ Failure Assessment (SOFA) score; nevertheless, a reduction in predicted versus observed mortality was also found in the control group. Table 2 summarizes the findings.

### Effect on length of stay in the intensive care unit

Five studies reported the effect on length of stay in the intensive care unit (ICU);^(9-11,18,19)^ however, only two of them^(10,18)^ (one RCT and one NRSI) reported standard deviation, so a quantitative synthesis was not performed. Only one NRSI^(18)^ found significant differences that favored the use of CytoSorb®.

### Effect on adverse events

Three studies reported adverse events,^(10,18,20)^ two reported no adverse effects,^(10,18)^ and the other reported one serious adverse event,^(20)^ namely a decreased platelet count, which was identified as probably related to the use of CytoSorb®. In this same study, 3 treatment discontinuations were reported in 3 patients during the study due to adverse events likely related to therapy.

## DISCUSSION

To our knowledge, this is the first meta-analysis evaluating the use of CytoSorb®, a hemadsorption device, in the setting of sepsis and septic shock, including clinical trials and cohort studies. Our study did not demonstrate a benefit of the use of CytoSorb® on mortality; however, it should be noted that the studies were heterogeneous, that the evidence for the RCTs was of high risk of bias, and that for the NRSIs, it was of moderate-to-severe risk of bias. Therefore, future research, of higher quality, could change or modify the direction of the effect.

Moderate heterogeneity was found in the RCTs (I2 = 57%), and high heterogeneity was found among the NRSIs (I2 = 70%), which could be explained by the different etiologies of sepsis, the severity of the disease, different kinds of interventions such as ECMO or continuous renal replacement therapy (CRRT) and the mode of use of the therapy. This heterogeneity makes it difficult to interpret a meta-analysis of these studies.

Two previous meta-analyses have evaluated the use of extracorporeal blood purification in sepsis;^(21,22)^ unlike our study, they did not focus on the use of CytoSorb® hemadsorption and did not include cohort studies; however, they included the same RCT,^(10,20)^ finding similar results. There is a recently published meta-analysis that evaluated the use of CytoSorb® in critically ill patients. This study found low-certainty evidence showing that the use of CytoSorb® might increase mortality; however, it did not find differences in adverse events.^(23)^

We found a significant decrease in the use of vasopressors in two studies,^(10,18)^ but we did not carry out a quantitative synthesis because they were of a different type. These findings are consistent with multiple quasiexperimental before-and-after studies that indicated that the use of CytoSorb® therapy resulted in decreased doses of vasopressors, hemodynamic stabilization, and improvement in metabolic parameters.^(2,24-27)^ Some studies reported that early use (within the first 24 - 48 hours), filtered blood volume, and prolonged duration of CytoSorb® therapy were associated with lower mortality;^(2,20,26,28-30)^ unfortunately, not all studies reported these variables.

Regarding the levels of inflammatory markers, it has been reported that CytoSorb® is effective in vitro for the elimination of both inflammatory and proinflammatory cytokines, as well as for a decrease in CRP and procalcitonin levels,^(3,31)^ and that the levels of cytokines correlate with both the severity of the disease and mortality.^(32,33)^ In this revision, only one study reported a nonsignificant decrease in IL-6,^(20)^ and two studies reported a decrease in the levels of CRP and procalcitonin;^(10,18)^ these findings coincide with before-and-after studies, not included in this review, where CytoSorb® was shown to reduce the levels of inflammatory markers.^(26,27,34,35)^

The predicted mortality based on the SOFA score was calculated in two studies; unfortunately, these findings could not be meta-analyzed due to a lack of data in one of the studies.^(11)^ A reduction between the observed *versus* predicted mortality was found in both studies. These findings are similar to those reported in other studies not included.^(30,36)^

CytoSorb® is considered to be a biocompatible and hemocompatible device,^(37)^ and studies in cardiac surgery and sepsis suggest that CytoSorb® does not induce coagulopathy, hemolysis, or clinically relevant side effects,^(26,38,39)^ which seems to coincide with the findings of the present review, where only one serious adverse event related to therapy was reported. However, it is worth mentioning that the use of CytoSorb® in the setting of sepsis is generally longer and that CytoSorb® may influence the elimination of or decrease in serum concentrations of some drugs; most of the time, CytoSorb® application requires interventions including extracorporeal membrane oxygenation (ECMO) and renal replacement therapy to be carried out. Therefore, as in other studies, we suspect that the adverse effects were underreported and not systematically evaluated.^(23)^

An important limitation is that meta-analysis was only conducted for the main outcome, and we did not perform it for secondary outcomes due to the small number of studies found or the lack of data. Studies were at moderate-to-high risk of bias, mainly due to confounding and study participant selection bias. It should be noted that the number of studies evaluating the use of CytoSorb® in sepsis and septic shock is limited.

Our review has other limitations. First, we did not include unpublished studies, conduct a search of the gray literature, or include conference abstracts or nonoriginal articles. Second, studies without a control group were not included since our main objective was to assess mortality. Third, the starting time, the duration of therapy, the volume of blood filtered, and the number of cartridges used were different or were not described in some studies, which could have affected the results. Fourth, only one study did not use renal replacement therapy (RRT), and two of them used it according to the patient’s needs, as it is known that acute renal failure can amplify the septic cascade induced by endotoxins, so the use of RRT could have affected the result. Fifth, our study focused only on short-term mortality.

## CONCLUSION

Our study found very low certainty evidence that shows no benefit of CytoSorb® use in terms of mortality at 28 - 30 days. We cannot recommend the use of CytoSorb® in septic or septic shock patients outside clinical trials. Further high-quality randomized trials with a common intervention arm are needed to evaluate the influence of CytoSorb® in this population.
